# Lipid Droplet-Associated Factors, PNPLA3, TM6SF2, and HSD17B Proteins in Hepatopancreatobiliary Cancer

**DOI:** 10.3390/cancers13174391

**Published:** 2021-08-31

**Authors:** Yoshiaki Sunami, Artur Rebelo, Jörg Kleeff

**Affiliations:** Department of Visceral, Vascular and Endocrine Surgery, Martin-Luther-University Halle-Wittenberg, University Medical Center Halle, 06120 Halle, Germany; artur.rebelo@uk-halle.de (A.R.); joerg.kleeff@uk-halle.de (J.K.)

**Keywords:** lipid droplets, pancreatic cancer, liver cancer, NAFLD, NASH, fibrosis, PNPLA3, TM6SF2, HSD17B, cancer-associated fibroblasts

## Abstract

**Simple Summary:**

Aberrant lipid synthesis and reprogrammed lipid metabolism are both associated with the development and progression of pancreatic and liver cancer. Most cells store fatty acids in the form of triacylglycerols in lipid droplets. Lipid droplets are intracellular organelles that not only store neutral lipids, but also play roles as molecular messengers and signaling factors. Some cancer cells accumulate massive amount of lipid droplets. Lipid droplets and lipid droplet-associated factors are further implicated to mediate proliferation, invasion, metastasis, as well as chemotherapy resistance in several types of cancer. This review dissected recent findings on the role of several lipid droplet-associated factors, patatin-like phospholipase domain-containing 3 (PNPLA3), Transmembrane 6 superfamily member 2 (TM6SF2), and 17β-hydroxysteroid dehydrogenase (HSD17B) 11 and 13 as well as their genetic variations in hepatopancreatobiliary diseases, especially cancer.

**Abstract:**

Pancreatic and liver cancer are leading causes of cancer deaths, and by 2030, they are projected to become the second and the third deadliest cancer respectively. Cancer metabolism, especially lipid metabolism, plays an important role in progression and metastasis of many types of cancer, including pancreatic and liver cancer. Lipid droplets are intracellular organelles that store neutral lipids, but also act as molecular messengers, and signaling factors. It is becoming increasingly evident that alterations in the regulation of lipid droplets and their associated factors influence the risk of developing not only metabolic disease but also fibrosis and cancer. In the current review article, we summarized recent findings concerning the roles of lipid droplet-associated factors, patatin-like phospholipase domain-containing 3, Transmembrane 6 superfamily member 2, and 17β-hydroxysteroid dehydrogenase 11 and 13 as well as genetic variants in pancreatic and hepatic diseases. A better understanding of cancer type- and cell type-specific roles of lipid droplet-associated factors is important for establishing new therapeutic options in the future.

## 1. Lipid Synthesis and Lipid Droplets in Pancreatic and Hepatic Cancer

Pancreatic cancer is currently the fourth leading cause of cancer deaths in the United States both in females and males [1]; primary liver cancer-related death positions as the seventh place in females and the fifth place in males reported in 2021 [1]. Pancreas and liver cancers are predicted to be the second and third most common cancer death by 2030, respectively [2]. Although recent therapeutic advance such as more effective palliative, adjuvant, and neo-adjuvant chemotherapy, the overall 5-year survival rate of pancreatic cancer is still 10% [1,3]. The overall 5-year survival rate for liver cancer is 20% [1]. Both dysregulated lipid synthesis and lipid metabolism reprogramming contribute to the development and progression of pancreatic cancer [4]. Nonalcoholic fatty liver disease (NAFLD) causes hepatocellular carcinoma (HCC) in 13%–38.2% of patients, unrelated to virus and alcohol [5].

**Figure 1:**
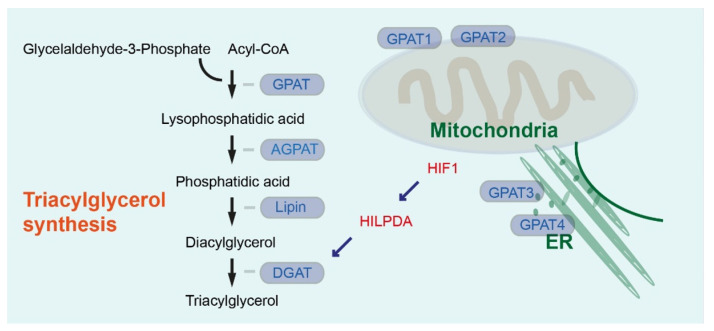
Substrates and key enzymes in triacylglycerol synthesis. AGPAT: Acylglycerolphosphate acyltransferase, DGAT: Diacylglycerol acyltransferase, ER: Endoplasmic reticulum, GPAT: Glycerol-3-phosphate acyltransferase.

For fatty acid (FA) synthesis, ATP-citrate lyase (ACLY) catalyzes the reaction of generation of cytoplasmic acetyl-CoA from citrate. Acetyl-CoA carboxylase (ACC) subsequently catalyzes conversion into malonyl-CoA. Multi-enzyme protein fatty acid synthase (FASN) converts malonyl-CoA and acetyl CoA coupled to the acyl carrier protein (ACP) domain into a basic 16-carbon saturated FA, palmitic acid [4,6,7]. On the cytosolic side of the endoplasmic reticulum (ER), longer FAs are produced. Several types of fatty acid desaturases such as Δ^9^-stearoyl-CoA desaturase (SCD) introduce carbon double bonds. SCD is the rate-limiting enzyme catalyzing mainly the synthesis of monounsaturated 16- or 18-carbons molecules palmitoleate and oleate from palmitoyl-CoA and stearoyl-CoA, respectively [4,6,7]. Most cells store FAs in the form of triacylglycerols (TAGs) in the lipid droplets (LDs). Glycerol-3-phosphate acyltransferase (GPAT) catalyzes the first step of synthesis of TAG, by the acylation of glycerol-3-phosphate and acyl-CoA to synthesize lysophosphatidic acid (LPA) (Figure 1) [8]. GPAT1 and GPAT2 are localized in the outer membrane of mitochondria. GPAT3 and GPAT4 are localized in the ER membrane [9]. It has been shown that GPAT4 re-localizes from the ER to a subset of forming LDs and mediates LD growth [10]. Expression of GPAT1 is high in liver and adipose tissues, GPAT3 plays an important role in TAG synthesis in white adipose tissue (WAT), and GPAT4 is required for the production of TAGs in the mammary gland [9]. GPAT1-deficient mice (global knockout, *Gpat1^−/−^*) have reduced susceptibility to hepatotoxin diethylnitrosoamine (DEN)-induced liver tumorigenesis [11]. GPAT2 is highly expressed in several human cancers such as melanoma, lung, prostate, and breast cancer [12]. *GPAT3* expression is associated with a shorter overall survival of endometrial cancer patients [13].

LPA is converted into phosphatidic acid (PA) via the acylglycerolphosphate acyltransferase (AGPAT) family (Figure 1). There are eleven known isoforms of AGPAT, each encoded by a different gene [14]. A comparative meta-profiling analysis of fourteen different tumor types (bladder, breast, central nervous system, colorectal, leukemia, lung, lymphoma, melanoma, mesothelioma, ovary, pancreas, prostate, renal, and uterus) identified increased expression of *AGPAT2* as a Heme Oxygenase-1 (HO-1) target gene [15]. Hypoxia-inducible factor 1 (HIF-1) also regulates the expression of AGPAT2. AGPAT2 promotes the survival of cancer cells under hypoxia [16]. On the other hand, lower *AGPAT9* expression is associated with significantly shorter overall survival of clear-cell renal cell carcinoma patients [17]. Furthermore, AGPAT9 suppresses breast cancer cell growth, invasion, and metastasis [18]. So far, the precise role of each AGPAT family member in hepatopancreatobiliary cancer has not been clarified.

PA phosphatase (also known as lipin) dephosphorylates PA to produce diacylglycerol (DAG). DAG is converted into TAG by diacylglycerol acyltransferase (DGAT) (Figure 1) [8]. *DGAT1* and *DGAT2* belong to two distinct gene families. It has been shown that patients with DGAT1 deficiency exhibit intestinal failure [19]. When DGAT1 function is deficient in patient-derived intestinal organoids, FAs cannot be incorporated in LDs, leading to lipotoxicity and cell death [20]. Hypoxia-inducible LD associated (HILPDA), which is regulated by HIF1, is known to regulate LD storage and promotes LD formation [21]. It has been further shown that nutrient deprivation post-transcriptionally upregulates HILPDA protein independent of HIF1 transactivation [22]. HILPDA preferentially accumulates in remodeling or expanding LDs. HILPDA has been shown to co-localize with DGAT1 and DGAT2 [21]. HILPDA induces DGAT1 and promotes lipid storage in hepatocytes [23]. The role of DGAT1 in LD storage and protection from lipotoxicity can also be tumor promoting. It has been shown that DGAT1 prevents lipotoxicity in glioblastoma by promoting LD storage of FAs. High levels of DGAT1 are associated with poor survival in patients with glioblastoma [24]. DGAT2 has been suggested to reduce HCC aggressiveness. High expression of DGAT2 is associated with longer survival of patients with HCC [25]. Another study suggested that obesity promotes gastric cancer metastasis via DGAT2-dependent accumulation of LDs [26]. In summary, several enzymes in the glycerolphosphate pathway involved in TAG synthesis and LD storage have been shown to be involved in several cancer types either positively or negatively. Yet, the precise role of DGAT1 and DGAT2 in hepatopancreatobiliary cancer has not been elucidated.

## 2. Role of Lipid Droplets and Lipid Droplet-Associated Factors in Hepatopancreatobiliary Cancer

LDs are ubiquitous intracellular organelles that store neutral lipids such as sterol esters and TAGs [27,28]. Cellular functions of LDs further include membrane synthesis, viral replication, and protein degradation [27]. LDs are composed of a phospholipid monolayer together with different types of proteins such as structural proteins, membrane transport proteins, and enzymes [29]. Some cancer cells accumulate large amount of LDs [28]. LDs are further implicated to mediate the proliferation, invasion, and metastasis, as well as chemotherapy resistance [29]. Oncogenic KRAS is the most important driver for pancreatic cancer development. Oncogenic KRAS controls LD homeostasis and supports reprogramming of tumor cell metabolism, invasion, and migration [30]. Oncogenic KRAS expression in combination with serpin family F member 1 (Serpinf1)-deficiency in mice (*Ela1-Kras^G12D/+^; Serpinf^−/−^*) increases matrix metalloproteinase 2 (MMP-2) and MMP-9 expression, peripancreatic fat with adipocyte hypertrophy, and intrapancreatic infiltration of adipocytes. SERPINF1, previously known as pigment epithelium-derived factor, PEDF, is a potent anti-angiogenic factor and more than half of pancreatic cancers have reduced levels of SERPINF1 [31]. Genetic ablation of *Serpinf1* increases cerulein-induced pancreatic inflammation and fibrosis in mice [32]. The stroma of mice with oncogenic KRAS and SERPINF1-deficiency demonstrates elevated expression of LD-associated proteins of perilipin family members PLIN2 (Adipophilin, also known as Adipose differentiation-related protein, ADRP) and PLIN3 (also known as TIP47), which is associated with increased adipogenesis, and decreased levels of patatin-like phospholipase domain-containing 2 (PNPLA2, also known as adipose tryglyceride lipase, ATGL) [31].

**Figure 2:**
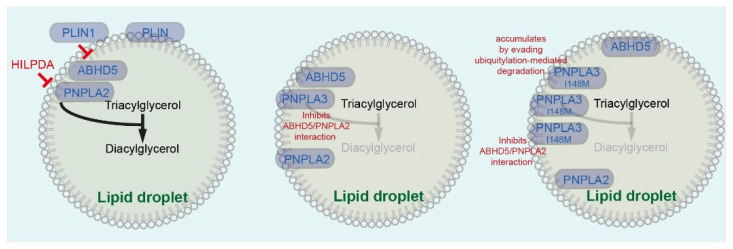
Lipid droplet-associated factors involved in lipolysis. PNPLA3 I148M variants sequesters ABHD5 that limits access to PNPLA2 and attenuates PNPLA2-mediated lipolysis. ABHD: Alpha beta hydrolase domain, HILPDA: Hypoxia-inducible LD associated, PLIN: Perilipin, PNPLA: Patatin-like phospholipase domain-containing.

PNPLA2 is a functional lipase involved in the lipolysis of TAGs [33]. Enzymatic action of PNPLA2 is regulated by its LD localization and interaction with its co-activator called alpha beta hydrolase domain 5 (ABHD5), also called comparative gene identification (CGI-58) (Figure 2) [34,35]. A deficiency in the lipolytic enzyme PNPLA2 may promote pancreatic steatosis by inducing an imbalance in TAG turnover and an increase in the storage of LDs [31]. On the contrary, another study showed that high expression of PNPLA2 is associated with adiposity and increased tumor stroma in patients with pancreatic cancer [36]. This study by Grace et al. suggested that PNPLA2 may increase free FA content in the tumor microenvironment and increase in-stromal proliferation [36]. The enzymatic activity of PNPLA2 is inhibited by HILPDA (Figure 2). Inhibition of enzymatic activity of PNPLA2 by HILPDA leads to inhibition of lipolysis, attenuated fatty acid oxidation and reactive oxygen species (ROS) production. Consistently, HILPDA has been shown to support intracellular lipid accumulation by enhancing triglyceride synthesis [21]. Upregulation of HILPDA has been observed in various cancer cells including renal cell carcinoma, ovarian clear cell carcinoma, colorectal adenoma, and carcinoma. Overexpression of *HILPDA* is associated with significantly shorter overall survival in pancreatic cancer patients [37]. HILPDA ablation impairs xenografted colorectal cell tumor growth in nude mice and TAG storage in tumors [22]. Yet, the precise role of HILPDA has not been fully elucidated either in pancreatic or liver cancer.

PLINs are the most abundant LD proteins and five *PLIN* genes encode five major PLIN proteins (PLIN1–5), which are discussed in detail elsewhere [38]. PLINs play an important role in regulating lipid storage, LD size, and in mediating organelle interactions [38]. PLIN1 is expressed in adipocytes, and most cells have LDs with PLIN2 and PLIN3 at the surfaces. PLIN5 controls lipolysis in oxidative tissue such as skeletal muscle, heart, and brown adipose tissue [35]. In HCC, PLIN1–3 are co-expressed. PLIN2 expression correlates with the proliferation rate and is upregulated during tumorigenesis, whereas PLIN1 is often lost during hepatocellular carcinogenesis [39]. Under basal conditions, PLIN1 binds ABHD5 and attenuates lipolysis (Figure 2) [35]. PLIN1 is a direct target gene of the Farnesoid X receptor (FXR). FXR upregulates PLIN1 to stabilize LDs and thereby prevents the activation of hepatic stellate cells (HSCs) [40]. FXR is a promising target of NASH, fibrosis, and cancer. Various FXR agonists have shown anti-fibrotic effects and are used in the treatment of chronic liver disease, hepatocellular cancer, and metabolic diseases [40,41]. PLIN2 protein expression is associated with shorter overall survival and early recurrence-free survival of pancreatic cancer [42]. PLIN2 knockdown activates unfolded protein response (UPR) signaling in clear-cell renal cell carcinoma (ccRCC) cells. PLIN2 promotes lipid growth and tumor growth in ccRCC xenografts [43]. On the other hand, it has been shown that high expression of the *PLIN2* gene is associated with longer overall survival and disease-free survival, but high expression of *PLIN3* is associated with shorter overall survival and disease-free survival of patients with ccRCC [44,45]. The role of each PLIN member seems to be cancer type-dependent.

## 3. PNPLA3 in Pancreatic and Hepatic Diseases

The human patatin-like phospholipase domain-containing 3 (PNPLA3), also known as adiponutrin (ADPN), is a LD-associated protein. PNPLA2 and PNPLA3 are encoded by close paralogues but appear to have opposite functions in TAG mobilization and storage. PNPLA3 attenuates ABHD5/PNPLA2 interaction and suppresses ABHD5/PNPLA2-dependent lipolysis (Figure 2) [46]. Sterol response element-binding protein-1 (SREBP-1) and liver X receptor (LXR) activate *PNPLA3* gene expression [47,48]. Chronic overexpression of PNPLA3 in the liver under the human *APOE* promotor with a hepatic enhancer sufficiently caused hepatic steatosis in a transgenic mouse model [49]. The variant rs738409 is a C > G substitution, leading to isoleucine (I) to methionine (M) substitution at position 148 (I148M) [50]. PNPLA3 I148M inhibits PNPLA2 in an ABHD5-dependent manner (Table 1) [51]. Mechanistically, PNPLA3 I148M sequesters ABHD5 that limits access to PNPLA2 and attenuates PNPLA2-mediated lipolysis (Figure 2). Furthermore, PNPLA3 I148M can accumulate on LDs by evading ubiquitylation-mediated degradation (Figure 2) [52]. Consistently, *Pnpla3^I148M/I148M^* knock-in mice accumulate PNPLA3 on LDs and develop hepatic steatosis [53]. Hepatocyte-specific overexpression of PNPLA3 I148M variant accelerates steatohepatitis and led to liver fibrosis in a diet-induced animal model of NAFLD that sequentially developed a fatty liver, steatohepatitis, and progression of fibrosis on a high-fat diet (HFD) with the provision of sugars in the drinking water (named DIAMOND mouse model) [54]. Human carriers of *PNPLA3* rs738409 show increased hepatic TAG content (Table 1) [55]. *PNPLA3* rs738409 genetic variation confers susceptibility to NAFLD, associated with hepatic fat content (Table 1) [56]. Carriage of the *PNPLA3* rs738409 C > G variant is associated with an increased risk of NAFLD-associated HCC (Table 1) [57]. The rs738409 genetic variant is associated with HCC in obese patients (Table 1) [58]. In the U.S. population without viral hepatitis, PNPLA3 I148M and higher NAFLD liver fat (metabolic syndrome, type 2 diabetes, fasting serum insulin, ALT, and AST were used as criteria) and fibrosis scores (age, BMI, impaired fasting glucose, diabetes, platelet count, and albumin were used as criteria) are associated with increased liver disease mortality (Table 1) [59]. The *PNPLA3* rs738409 C > G variant is associated with the severity of liver fibrosis and fibrosis progression in patients with NAFLD (Table 1) [60,61]. The *PNPLA3* rs738409 C > G variant is not associated with alcoholic chronic pancreatitis (Table 1) [62]. Yet, it has not been clarified whether *PNPLA3* rs738409 genetic variation is associated with the incidence or prognosis of pancreatic cancer patients.

## 4. PNPLA3 in Stellate Cells and Cancer-Associated Fibroblasts

PNPLA3 is highly expressed in hepatic stellate cells (HSCs) [64]. Expression of PNPLA3 is induced during the activation of HSCs. Knockdown of *PNPLA3* by siRNA in HSC cells is associated with a reduction of α-smooth muscle actin (α-SMA), suggesting that PNPLA3 is positively associated with HSC activation [76]. Overexpression of wild-type PNPLA3, but not the I148M variant, in human HSC cells induces a reduction in the secretion of MMP-2, tissue inhibitor of metalloprotease 1 and 2 (TIMP1 and TIMP2) [77]. Incubation with insulin leads to PNPLA3 upregulation and higher expression of wild-type PNPLA3 promotes a reduction of LD content in HSC cells [64]. Retinoids are stored as retinyl esters in the retina and stellate cells. PNPLA3 exhibits retinyl-palmitate lipase activity in human HSCs. The wild-type PNPLA3 hydrolyzes retinyl palmitate into retinol and palmitic acid in HSCs [64]. On the contrary, retinyl-palmitate lipase activity is reduced in the PNPLA3 I148M variant. Therefore, overexpression of PNPLA3 I148M does not lead to the reduction of LD content in HSCs [64]. Consistently, homozygous *PNPLA3* rs738409 carriers show elevated retinyl-palmitate storage in the liver [55]. HSCs carrying the PNPLA3 I148M variant retain less retinol content, leading to reduced retinoid X receptor (RXR)/retinoic acid receptor (RAR) transcriptional activity [76]. Human HSCs carrying the *PNPLA3* rs738409 variant further show a reduced LXRα expression and transcriptional activity compared with wild-type HSCs (Table 1) [63]. Expression of de novo lipogenic genes such as fatty acid synthase (*FASN*), Δ^9^-stearoyl-CoA desaturase 1 (*SCD1*), and sterol regulatory element binding transcription factor 1c (encoded by *SREBF1* gene) is decreased in primary as well as in the PNPLA3 I148M variant overexpressing HSCs [63]. Expression of the master transcriptional regulator of the mevalonate pathway, SREBP-2 (encoded by the *SREBF2* gene), is reduced in PNPLA3 I148M variant-overexpressing HSCs [63].

*PNPLA3* rs738409 C > G homozygotes have lower circulating levels of retinol-binding protein 4 (RBP4) (Table 1) [64]. RBP4 facilitates the transport of retinol from the liver to peripheral organs [78]. In hepatocytes, retinyl esters are converted to retinol and subsequent binding to RBP4 stimulates the release of the retinol-RBP4 complex to the circulation (Figure 2) [79]. Hepatic retinol and RBP4 levels are reduced, but retinyl-palmitate levels are elevated in mice fed a high-fat, high cholesterol diet and *Leptin^ob^* mutant (*ob/ob*) mice, which are considered as mouse NAFLD models [79]. Retinoids are natural and synthetic vitamin A derivatives such as all-trans retinoic acid (ATRA), *9-cis* retinoic acid (9-RA), and *13-cis* retinoic acid (13-RA). Retinoids are considered to control cellular differentiation, growth, and apoptosis [80]. Retinol and its metabolites, ATRA and 9-RA, induce quiescence of activated pancreatic stellate cells (PSCs) [81,82]. Incubation with ATRA in HSC cells overexpressing the PNPLA3 I148M mutant protein leads to reduction in extracellular protein levels of MMP-2, TIMP1, and TIMP2 [77]. Quiescent PSCs produce Secreted Frizzled Related Protein 4 (SFRP4). SFRP4 inhibits Wnt-meditated signal transduction by sequestering Wnt molecules [82]. It has been shown that the aberrant of Wnt-β-catenin signaling promotes the development and/or progression of liver cancer [83]. Wnt signaling promotes the initiation and progression of pancreatic cancer [84]. ATRA administration leads to a reduction of activated stroma as well as the reduction of cancer cell proliferation in a mouse model called KPC (*Pdx1-Cre; lox-stop-lox-Kras^G12D/+^; lox-stop-lox-Trp53^R172H/+^*) [82]. The KPC mouse develops widely metastatic pancreatic cancer with a predominantly glandular histology [85]. Further ATRA treatment increases apoptosis of cancer cells associated with a decrease in nuclear β-catenin and increase in stromal SFRP4 in KPC mice [82]. Knockdown of *SFRP4* in PSCs increases the nuclear β-catenin [82], suggesting that stromal SFRP4 may induce cancer cell death and may inhibit Wnt-β-catenin signaling. However, on the contrary, another study showed that the expression of SFRP4 was increased in pancreatic cancer lesions in KPC mice and a high expression of SFRP4 was observed in tumor lesions of pancreatic cancer patients. Furthermore, high expression of SFRP4 in the serum and tumor lesions is correlated with shorter overall survival of pancreatic cancer patients [86]. It might be possible that the role of SFRP4 in pancreatic cancer is different between in PSCs and in epithelial cells. The precise cell type-specific role of SFRP4 in pancreatic cancer still need to be elucidated. The aberrant of Wnt-β-catenin signaling supports not only pancreatic and liver cancer development, but also metabolic disease. A genome-wide association study confirmed that *PNPLA3* is a risk factor for the full histological spectrum of NAFLD at genome-wide significance levels, but also suggested that the Wnt signaling pathway may be relevant in NAFLD pathogenesis [87]. So far, it is not clear whether there is a direct association between PNPLA3 genetic status and Wnt signaling activation or not.

HSCs isolated from patients with NASH carrying the PNPLA3 I148M variant produce and release higher amounts of the chemokine CCL5 and granulocyte-macrophage colony-stimulating factor (GM-CSF) than HSCs with wild-type PNPLA3. Overexpression of the PNPLA3 I148M variant in human HSCs leads to higher secretion of chemokines and cytokines such as CCL2, CCL5, CXCL1, CXCL8, and GM-CSF [63,76]. Mesenchymal stem cells (MSCs) are a major source of CAFs. Cancer-associated MSCs (CA-MSCs) secrete GM-CSF. CA-MSC-derived GM-CSF is required for pancreatic cancer cell invasion and metastasis [88,89]. Therefore, it is considerable that stromal PNPLA3 I148M variant assists pancreatic cancer metastasis.

So far, drugs specifically targeting the PNPLA3 I148M variant have not been identified. However, there are several clinical trials for patients with the PNPLA3 I148M variant. One study (NCT04640324) aimed to explore the effect of the silybin-phospholipid complex, vitamin D, and vitamin E in NAFLD patients carrying *PNPLA3* rs738409, *TM6SF2* rs58542926, and Membrane Bound O-Acyltransferase-Domain-Containing 7 (*MBOAT7*) rs641738 genetic variants. The outcome of the study has not been published. Another study tested whether the *PNPLA3* rs738409 variant affects the response to diet rich in ω-3 polyunsaturated fatty acid (PUFA) (e.g., fish and nuts) in obese adolescents (NCT01556113). A diet rich in PUFA improved fatty liver disease in obese adolescents, and patients with the *PNPLA3* rs738409 variant tended to have a better response to the dietary intervention [90]. A phase 1 study currently recruits patients with NASH and the *PNPLA3* rs738409 variant to investigate the safety and tolerability of AZD2693, a drug which structure and role have not been disclosed (NCT04483947).

## 5. TM6SF2 Variant in NAFLD, Fibrosis, and Cancer

An exome-wide association study of liver fat content identified that Transmembrane 6 superfamily member 2 (TM6SF2) rs58542926 C > T polymorphism (E167K). TM6SF2 activity is required for the secretion of VLDL and impaired TM6SF2 function leads to NAFLD (Table 1) [65]. Consistently, hepatocyte-specific deletion of TM6SF2 (*Alb-Cre*; *Tm6sf2^lox/lox^*) impaired VLDL secretion and promoted steatosis [91]. The *TM6SF2* rs58542926 E167K variant is associated with increased circulating TAGs in patients with NAFLD (Table 1) [66], as well as hepatic TAG content [67]. The *TM6SF2* rs58542926 E167K variant is also associated with hepatic fibrosis and cirrhosis, independent of potential cofounding factors such as body mass index (BMI), type 2 diabetes mellitus, and the *PNPLA3* rs738409 genotype (Table 1) [68]. Consistently, hepatocyte-specific deletion of TM6SF2 (*Alb-Cre*; *Tm6sf2^lox/lox^*) promotes fibrosis [91]. A genome-wide association study (GWAS) identified *TM6SF2* as risk loci for alcohol-related cirrhosis [92]. Hepatocyte-specific deletion of TM6SF2 enhanced liver tumorigenesis in two HCC models, either neonatal mice injected with Streptozotocin and high fat-fed or with diethylnitrosamine (DEN) injection plus high trans-fat fructose (TFF) diet feeding [91]. In univariate but not multivariate analysis, homozygote carriage of the *TM6SF2* rs58542926 C > T polymorphism is associated with an increased risk of NAFLD-HCC (Table 1) [68]. So far, there is a study showing that TM6SF2 suppresses HSC activation [93], but there has been until now no significant study addressing the role of TM6SF2 in pancreatic diseases.

## 6. HSD17B11 and HSD17B13 in Pancreatic and Liver Cancer

17β-hydroxysteroid dehydrogenases (HSD17Bs) catalyze the conversion between 17-keto- and 17-hydroxysteroids [94]. In humans, so far 15 HSD17B members have been identified [94]. HSD17B13 is a LD-associated protein, which is mainly restricted to the liver [95]. HSD17B13 is located on the surface of LDs and its expression is upregulated in the livers of LD fraction in fatty liver of patients as well as in steatotic livers of *db/db* (diabetic) mice and mice fed with a HFD [96]. Hepatic expression of *HSD17B13* is upregulated in NASH patients compared with controls [70]. It has been shown that HSD17B13 functions as a retinol dehydrogenase (RDH) associated with NAFLD [70]. HSD17B13 deficiency induces hepatic steatosis in male mice. The expression of key proteins in FA synthesis such as FASN, ACC1, and SCD1 is increased in the livers of *Hsd17b13* knockout mice [97].

HSD17B13 expression is induced by LXRα through SREBP-1c [98]. A GWAS study revealed that a genetic variant near *HSD17B13* (rs4607179) is associated with lower risk of alcohol-associated liver cirrhosis (Table 1) [69]. Another GWAS study identified that rs6834314 A > G variant near the *HSD17B13* gene is associated with increased steatosis and NAFLD histology (Table 1) [70]. Loss of the function variant of *HSD17B13* (rs72613567, an insertion of an adenine) is associated with reduced levels of alanine aminotransferase (ALT) and aspartate aminotransferase (AST) as well as of progression from steatosis to steatohepatitis [71]. The *HSD17B13* rs72613567 variant increases phospholipids and protects against fibrosis in NAFLD (Table 1) [72]. The *HSD17B13* variant protects from HCC development in alcoholic liver disease [73], and also reduces the risk of developing cirrhosis and HCC in alcohol misusers [74]. In another study, it was consistently shown that the rs72613567 variant of *HSD17B13* is associated with reduced risk of cirrhosis and HCC (Table 1). The insertion-allele is associated with a 33% lower rates of liver related mortality in the general population in Denmark and with up to 49% lower liver-related mortality in patients with cirrhosis [75]. The genetic variant in *HSD17B13* nonsynonymous SNP rs62305723 G > A encodes a P260S substitution. The protein HSD17B13 P260S retains LD localization but lacks RDH activity. The *HSD17B13* rs62305723 G > A variant is associated with decreased ballooning and inflammation (Table 1) [70].

Another HSD17B family member, HSD17B11, is 78% homologous to HSD17B13. Genes for both enzymes are located in same chromosomal locus in humans, mice, and rats, suggesting a coevolution of these genes [99]. HSD17B11 is known to convert 5α-androstane-3α, 17β-diol (3α-diol) to androsterone [100]. The *HSD17B11* rs9991501 T > C variant is associated with lean body mass, consisting mostly of skeletal muscle [101]. The variant in the *HSD17B11* locus was associated with the *HSD17B11* gene expression in skeletal muscle [101]. Exogenous expression of HSD17B11 induces LD aggregation and elevated TAG levels. Further, exogenous expression of HSD17B11 results in a reduction of PNPLA2 on LDs without changing the localization or abundance of PLIN2 [102]. Interestingly, elevated expression of *HSD17B11* is associated with shorter overall survival of pancreatic cancer patients [37]. The precise role of HSD17B11 in pancreatic cancer still needs to be elucidated.

## 7. Conclusions

Lipid metabolism is implicated not only in metabolic diseases, but rather in a broad range of diseases such as fibrosis and cancer. LD-associated proteins play an important role in dynamics of LD and it is now evident that expression or genetic variation of several LD-associated factors are associated with overall survival in pancreatic cancer patients. The PNPLA3 variant is associated with steatosis, fibrosis, and liver cancer. The PNPLA3 I148M sequence variant markedly increases the risk of disease progression. The TM6SF2 E167K variant is associated with NAFLD, fibrosis, and cirrhosis, as well as NAFLD-associated cancer. On the other hand, the loss of function of HSD17B13 caused by genetic variations has positive effects in hepatic diseases, and HSD17B11 could be a target for the treatment of pancreatic diseases. Taken together, genetic associations highlighted that lipid metabolism and LDs have a major impact on the pathophysiology of hepatopancreatobiliary diseases. Further studies are required to clarify how genetic variations of LD-associated factors alter downstream signaling events. Furthermore, it is important to increase our understanding of cancer-type-specific as well as cell-type-specific roles of LD-associated factors, which may help to develop more specific and personalized therapies for pancreatic and liver cancer patients in the future.

## Figures and Tables

**Table 1:** Genetic variations of *PNPLA3*, *TM6SF2*, and *HSD17B13* and phenotypical descriptions.

Gene	Variant	Amino Acid Change	Functional Relevance/Phenotypical Change	Reference
*PNPLA3*	rs738409 C > G	I148M	Inhibits PNPLA2	[51]
*PNPLA3*	rs738409 C > G	I148M	Increased hepatic TAG content	[55]
*PNPLA3*	rs738409 C > G	I148M	Confers susceptibility to NAFLD, associated with hepatic fat content	[56]
*PNPLA3*	rs738409 C > G	I148M	Increased risk of NAFLD-associated HCC	[57]
*PNPLA3*	rs738409 C > G	I148M	Associated with HCC in obese patients	[58]
*PNPLA3*	rs738409 C > G	I148M	Associated with increased liver disease mortality	[59]
*PNPLA3*	rs738409 C > G	I148M	Associated with the severity of liver fibrosis and fibrosis progression in patients with NAFLD	[60,61]
*PNPLA3*	rs738409 C > G	I148M	Not associated with alcoholic chronic pancreatitis	[62]
*PNPLA3*	rs738409 C > G	I148M	Reduced LXRα expression and transcriptional activity in HSCs	[63]
*PNPLA3*	rs738409 C > G	I148M	Homozygotes have lower circulating levels of RBP4	[64]
*TM6SF2*	rs58542926 C > T	E167K	Impaired function contributes to NAFLD	[65]
*TM6SF2*	rs58542926 C > T	E167K	Associated with increased circulating TAGs in patients with NAFLD	[66]
*TM6SF2*	rs58542926 C > T	E167K	Associated with increased hepatic TAG content	[67]
*TM6SF2*	rs58542926 C > T	E167K	Associated with hepatic fibrosis and cirrhosis, increased risk of NAFLD-HCC	[68]
Near *HSD17B13*	rs4607179 A > C		Associated with lower risk of alcohol-associated liver cirrhosis	[69]
Near *HSD17B13*	rs6834314 A > G		Associated with increased steatosis and NAFLD histology	[70]
*HSD17B13*	rs72613567 A insertion		Associated with reduced levels of ALT, AST, reduced risk of chronic liver disease and of progression from steatosis to steatohepatitis	[71]
*HSD17B13*	rs72613567 A insertion		Increases phospholipids and protects against fibrosis in NAFLD	[72]
*HSD17B13*	rs72613567 A insertion		Protects from HCC development in alcohol liver disease	[73]
*HSD17B13*	rs72613567 A insertion		Reduces the risk of developing cirrhosis and HCC in alcohol misusers	[74]
*HSD17B13*	rs72613567 A insertion		Reduced risk of cirrhosis and HCC	[75]
*HSD17B13*	rs62305723 G > A	P260S	Retains LD localization but lacks RDH activity, decreased ballooning and inflammation	[70]

ALT: Alanine aminotransferase; AST: Aspartate aminotransferase; HCC: Hepatocellular carcinoma; HSD: Hydroxysteroid; LD: Lipid droplet; NAFLD: Nonalcoholic steatohepatitis; PNPLA: Patatin-like phospholipase; RDH: Retinol dehydrogenase; TAG: Triacylglycerol; TM6SF2: Transmembrane 6 superfamily member 2.
